# Internal quality control – past, present and future trends

**DOI:** 10.1515/almed-2022-0029

**Published:** 2022-05-23

**Authors:** Carmen Ricós, Pilar Fernandez-Calle, Carmen Perich, James O. Westgard

**Affiliations:** External Quality Programs Committee and Analytical Quality Commission, Spanish Society of Laboratory Medicine (SEQC^ML^), Barcelona, Spain; Department of Laboratory Medicine, La Paz University Hospital, Madrid, Spain; University of Wisconsin School of Public Health, Madison, WI, USA

**Keywords:** control with patients results, internal quality control, performance specifications

## Abstract

**Objectives:**

This paper offers an historical view, through a summary of the internal quality control (IQC) models used from second half of twentyth century to those performed today and wants to give a projection on how the future should be addressed.

**Methods:**

The material used in this work study are all papers collected referring IQC procedures. The method used is the critical analysis of the different IQC models with a discussion on the weak and the strong points of each model.

**Results:**

First models were based on testing control materials and using multiples of the analytical procedure standard deviation as control limits. Later, these limits were substituted by values related with the intended use of test, mainly derived from biological variation. For measurands with no available control material methods based on replicate analysis of patient’ samples were developed and have been improved recently; also, the sigma metrics that relates the quality desired with the laboratory performance has resulted in a highly efficient quality control model. Present tendency is to modulate IQC considering the workload and the impact of analytical failure in the patent harm.

**Conclusions:**

This paper remarks the strong points of IQC models, indicates the weak points that should be eliminated from practice and gives a future projection on how to promote patient safety through laboratory examinations.

## Introduction

Laboratory medicine is a discipline that provides information on patient health status and there is no doubt that many medical decisions are based on laboratory tests results; however, it is not objectively measured how they could impact on every patient healthcare [1].

In any case, laboratory professional should assure reliability of the information produced so that clinicians can avoid erroneous decisions that would adversely affect patient healthcare.

Daily laboratory work includes examination and extra-examination activities, whose quality has been extensively studied in our setting [2], [3], [4], [5], [6], [7]. The Spanish Society of Laboratory Medicine (SEQC^ML^) dedicates three working groups, namely Analytical Quality Commission and Extra-analytical Quality Commission, together with the External Quality Assurance Programs Committee, to implement best practices. (www.seqcinternational.com/en/society/management-council/_id:7/).

The examination phase of the global laboratory process is focused on the measurement of the biological substances that constitute human fluids. In this article a biological quantity appraised in a human fluid is termed as “measurand”. To implement a quality assurance system of the examination procedures used in the laboratory is an essential task. Two key activities are the implementation of internal quality control strategies and participation in external quality control programs to ensure the reliability of results [accurate and precise]. Other elements of the quality system, such as monitoring key performance indicators, internal audits, etc., are not considered in this paper.

The aim of internal quality control is to monitor the examination process in order to avoid producing erroneous information concerning patient health status.

This paper offers an historical view, through a summary of the internal quality control (IQC) models used from second half of 20th century to those performed today and wants to give a projection on how the future should be addressed.

## Materials and methods

The material used in this study are all papers collected referring IQC procedures.

The method used is the critical analysis of the different models used with a discussion on the weak and the strong points of each model.

## Results and discussion

### Bases from the past

The oldest model, developed in the 50s was based on a statistical criterion, from calculating the mean of a number of results from a single measurand in the same control sample. It was supported by the use of a control chart that showed the results of control samples plotted in the x-axis vs. time or day on the x-axis. The mean and standard deviation were marked on the y-axis. Control limits were defined as ±2 standard deviations from the mean, which meant that 95% of results were expected to fall within these limits and be “in-control” and the other 5% were considered to identify “out-of-control” situations [8, 9].

In the 80s James Westgard evaluated this approach in the context of multitest automated systems for multiple levels of control results at different concentration levels (usually within and outside the biological reference interval) for each measurand. Use of 2SD control limits was seen to cause a high level of false rejections. Alternate control rules were proposed to limit false rejections while at the same time maximizing error detection through application of multiple rules. Control rules were identified in shorthand abbreviations, such as 1_3s_ which indicates a run rejection when 1 control measurement exceeded control limits defined as the mean ± 3 SD. A series of rules 1_3s_/2_2s_/R_4s_/4_1s_/10_*x*_ were introduced as an example of a multirule [10, 11]. The individual rules were selected to maintain a low probability for false rejection (p_fr_) while the additive effect of the rules increased the probability for error detection (p_ed_). This multirole algorithm was implemented on many automatic analyzers, allowing the laboratory professional to select the operative control rules to be applied.

At this time, all IQC applications would be properly described as statistical process control (SPC). The quality required for the intended medical use of test results was not considered in the design or planning of SPC applications. Control limits were based on the observed variability of the testing process, without any connection to the clinical use of the test. Establishing that a testing method was acceptable for intended medical use depended on performing a proper “method evaluation” prior to implementation of the method. The concept of “allowable Total Error” had been introduced as the form of quality requirement for method evaluation and development [12].

The main weakness of this approach was that, by relying on a purely statistical criterion, the margins of tolerance were in line with the analytical performance inherent in the method. Additionally, it was not possible to make an assessment of the need to improve the performance of the different analytical methods because it was not possible to know to what extent the analytical performance of the different methods met the clinical needs.

Another weakness was that laboratories with different analytical performance used different control limits, which did not promote harmonization.

During the 80s to mid-90s, efforts to define quality requirements and link them to quality control were led by Professor C.-H. de Verdier from Sweden and Professor Mogen Horder from Denmark under the auspices of NORDKEM, Nordic Clinical Chemistry Projects that involved all the Scandinavian countries [13], [14], [15], [16].

In the second half of 90s a multinational group of experts, under the auspices of the *Standards Measurement and Testing Program* proposed a series of recommendations for IQC [17] that are summarized as the following:

– IQC should be integrated into the quality management system of the laboratory.
– IQC should be combined with an external program with target values traceable to higher order reference standards.
– IQC should assure that performance specifications based on biological variation were attached, whenever possible.
– For a measurement procedure with high error frequency, the IQC protocol should have the maximum p_de_ together with the lowest p_fr_ possible, to stimulate a good resolution and prevention of problems. The general tendency should be to use a relaxed operative rule with the minimum p_fr_ possible
– Patient results of all measurands requested should be reviewed before releasing tests results, but in any case, this revision substitutes control sample testing.
– It is better to prevent errors instead than to correct them.

The recommendations from Hyltoft et al. [17] introduced the concept of quality specifications based on biology and clinical use of laboratory reports instead of based on statistics, as had been done before. This approach had in mind the final use of medical laboratory measurements for patient care.

A hierarchic strategy concerning performance specifications was established in the Stockholm international consensus conference, in 1999; so, from that moment the quality specifications were calculated accordingly [18]. In this conference a Biologic Variation database, prepared by the SEQC^ML^ Analytical Quality Commission was presented [19]. It had been prepared by a compilation of published papers, excluding those with poor reliability and calculating the median value for each measurand. A list of quality specifications for bias, imprecision and total an analytical error was also included. This data base was biannually updated until 2014 and was universally well-known by its inclusion on the Westgard’ website [20].

A different concern that was raised in the 2000s was the commutability of control materials. Commercial controls generated by the *In Vitro* Diagnostic (IVD) providers, required stabilization, additives to adjust the desired concentrations, etc., so the materials could behave differently than real patients’ specimens when analyze by the routine laboratory methods. Soon, certain organizations used control samples of human origin simply frozen, maintained at −80 °C and distributed in aliquots without any other manipulation, which avoided the mentioned limitation [21]. The problem in this case was (and still is today) the difficulty to prepare the necessary amount of control material for daily IQC and the cost of maintenance at −80 °C.

For those measurands without stable control material available, it was recommended to develop patient-based QC algorithms, such as average of normals (AoN). The idea, first described in the 1960s, was to calculate the mean (or median) of a number of daily patient results and to monitor how this mean varied with time (moving average) [22]. The condition to apply this model was that measurands had to tight biologic control (such as electrolytes, calcium), or stable patient populations such as red cell indices [23]. AoN was not as useful for those analytes having wide population distributions. To provide guidance for when to use AoN algorithms, Cembrowski [24] utilized the ratio of the population standard deviation (SD) divided by the measurement SD in a nomogram to estimate the number of patient samples that would be needed for effective error detection.

### Present situation

Once in the XXI century IQC, models based on the analytical procedure performances were developed; the rationale was the sigma metrics, based on the relationship between the performance specification that should be attained, expressed in terms of total analytical allowable error (TE_a_, %) and the real performance, expressed in terms of systematic error in absolute values (abs SE, %) and imprecision (CV, %):

$$\text{Sigma }=\left({\text{TE}}_{\text{a}}-\text{absSE}\right)\text{/CV}$$

The higher the Sigma the more relaxed the IQC procedure can be, and *vice versa* [25].

If the IQC is focused on a single analytical procedure in a single laboratory, the term SE can be set to zero if the aim is to detect changes in the laboratory’s regular performance of the measurement procedure that could imply an inappropriate medical impact. However, when two or more analytical systems are used in the same or various laboratories of a single health care service, the SE has to be considered [26]. Moreover, even on a single analytical procedure, for long-term processes the use of different calibrator or reagent lots could produce SE, and this could cause problems in patient monitoring (i.e., PSA testing after radiotherapy as treatment of prostatic cancer eradication).

The tree key points of an IQC protocol are described in the following sections.

#### Key point 1. Analytical performance specifications

Total allowable error should be based on one of the three proposals from the Milan 1st IFCC Strategic Conference [27]. Ideally analytical performance specifications should be based on the impact of analytical error on patient care; however, it is difficult to establish the direct relationship between these two facts [26].

This is the reason why biological variation is more widely used basis to establish analytical performance specifications, because it assures the correct clinical use of lab tests. For measurands without data on biological variation or with weak physiological regulation, the state of the art (highest level of analytical performance technically achievable) is another option accepted in the Milan conference [27, 28].

Biological variation (BV) data are presently available at the EFLM website [29]. This new database has been created from an exhaustive revision of papers included in the first database [19, 20] as well as from the application of an effective bibliographic search, able to recover as many as BV papers possible. The EFLM BV Working Group has developed an evaluation tool with very strict criteria for evaluating the reliability of methodology used in the published papers and with a further meta-analysis to improve the robustness and reliability of the new BV estimates [30].

State of the art can be obtained from the external quality assessment program in which the laboratory participates, on the basis of the 20 or 30 percentiles of participants’ deviations [31]. The annual revision of the SEQC^ML^ programs shows 20, 30, 50, 70 and 90 percentiles for each measurand [https://www.contcal.org/qcweb/Qcw_Documentacio.aspx].

#### Key point 2. Performance of examination procedure

The basic performance of the examination procedure is measured in each laboratory under routine conditions, in terms of CV and systematic error (SE) that is the percentage deviation to the target value of control material, for each measurand. When using control materials provided by the manufacturer of the analytical system, it is recommended to calculate CV and target value with a minimum of 10–20 measurements of control material during 10–20 consecutive days, in analytical stable conditions (same reagents, same technician, etc.) also it is recommended to update this information after a longer period (6–12 months) [26]. It is good practice to assess the agreement of observed means with the range of target values defined by the provider on the same lot of control materials over the time of use.

Concerning control material, using stabilized controls independent of the analytical system provided by another manufacturer, the so-called *third-party controls,* is highly suggested to verify that the metrological procedure do not deviates from the target value of control material; the ISO 15189 [32] standard specifically recommends this type of controls. The target value is considered to be the average of results obtained by the peer group of laboratories [formed by those using same analytical principle, same method, same instrument and same control material lot].In this way, the central value to which the individual deviation is compared is statistically robust. This method helps evaluate the harmonization among labs, although not standardization because there is no traceability to any higher order reference standards [33, 34].

Indirectly this material gives a general view of deviation with respect to other laboratories if results from all labs are available, in same way that happens in an external program. A tendency exists to call this type of control as “internal-external control”; however, this term cannot be considered correct because they are not blind control samples, which is one of the main characteristics of external control. So, if this type of control is used as IQC, it has to be complemented with an external program.

Due to the fact that IQC should verify not only the measurement procedure reproducibility but also the bias with respect to target value, some authors insist to incorporate the concept of traceability to higher order standards by using commutable control materials with values assigned by certified reference methods [35, 36]. However, this idea may be quite difficult to do in practice because of the poor availability of these traceable and commutable controls materials for daily work.

For this reason, it is very important to participate in an external quality assessment control program using this type of control materials. Being a discontinuous task, it is logistically and economically affordable. These materials can be considered as reference standards and allows evaluating the accuracy of laboratory tests results [37, 38] and also the standardization among laboratories. 

#### Key point 3. IQC based on patients’ results

An alternative way to avoid the commutability problem is using patients’ results as a complementary IQC protocol.

It has been previously mentioned the AoN with the use of a moving average calculation (Hoffman, Bull) [22, 23], this model has been very much advanced in the last years, using the presently available laboratory informatics systems [39, 40]. Recommendations on verification and validation of an IQC protocol using the moving average before being implemented in the laboratory, has been recently published by the IFCC [41].

With the aim to increase the error detection power of this model, it is important to define the patients’ population included in the average; to do this, a previous multivariate regression analysis that includes the mean of previous 2000 patient results and other independent variables such as age, sex, outpatient vs. inpatient status, as well as the patients’ diagnostics and hospital service has been studied [42, 43].

On January 2022 the SEQC^ML^ Analytical Quality Commission has given a Continuous Educational course with a depth revision of this strategy, with emphasis on the factors that optimize its implementation [44]. These are:

– Definition of truncation limits to select the patient’s results to be averaged.
– Description of the algorithm to be used [mean, median, weighted mean, etc.
– Designation of the control limits, which have to be in accordance with the analytical performance specifications pretended by the laboratory.

A recent paper from Bayat et al. [45] compares the classic IQC model based on control materials (multirules) with the one based on patients’ results. They noticed that classic method is very practical in high sigma (sigma>4) procedures (simpler and quick to release patient’ results), whereas a multirule coupled with a simple moving average can improve error detection in low sigma procedures (sigma<4), in spite of its complexity.

The weak point of this approach is that it does not directly estimate analytical bias because patients’ results are unknown although it detects a change of bias, which is useful to monitor the procedure performance. This limitation would be obviated by combining the moving average IQC with an external quality control adequate for verifying laboratory bias.

It is possible to make daily duplicated analysis of various patient samples, to calculate the differences between duplicates and to obtain the so-called *Patient Delta* for each measurand. The Average of Delta ideally is zero and when it moves, a change of analytical bias is denoted [46]. Cervinski and colleagues applied this model to the laboratory informatics system for inpatients and they have investigated the number of the optimum patient sample pairs to attain maximum power detection for measurement bias [42].

Analytical imprecision, expressed in terms of standard deviation (SD), can be calculated from differences between duplicates using the formula:

$$\text{SD}={\left(\sum {\left(x_{2}-x_{1}\right)}^{2}/2n\right)}^{1/2}$$

where *x*_2_ and *x*_1_ are second and first result of each patient pair of results and *n* is the number of duplicates tested.

An excellent example of a large focus IQC based on patients’ results are the *Empower Project* of Linda Thienpont and coworkers [47]. A number of patient’s results coming from different laboratories are compiled together with records of analytical method, instrument, as well as calibrator and reagent lots used. Robust statistic parameters are obtained to give manufacturers and laboratories a realistic view on assay quality and comparability, as well as stability of performance or reasons for increased variation.

From the 2011 the concept of patient risk management is being integrated into the IQC protocol. The Clinical and Laboratory Standards Institute (CLSI) indicates that three risk factors have to be considered [48, 49]. These are:

– How likely the measurement procedure it is for the failure to occurs (probability).
– How severe the potential harm to the patient is if the failure goes undetected (severity).
– How reliably the IQC strategy can detect the failure if it occurs (detectability).

For this reason, the IQC protocol has to establish the frequency of control material testing and the number of controls per run. This depends on the number of patient samples to be tested, the control levels available, the measurement procedure imprecision and the maximum expected number of erroneous patient results reported owing to the occurrence of an out-of-control event [it is not the same to assume a 10% of misclassified patients for cardiac troponin, that’s is the key diagnostic test for myocardial infarction than for triglycerides or ALT, for example].That is, it is important to take into account the frequency of control testing to avoid delivering patient results from incorrect analytical runs [50], [51], [52].There are some informatics applications that could help medical laboratories to implement this type of IQC system.

**Figure 1:**
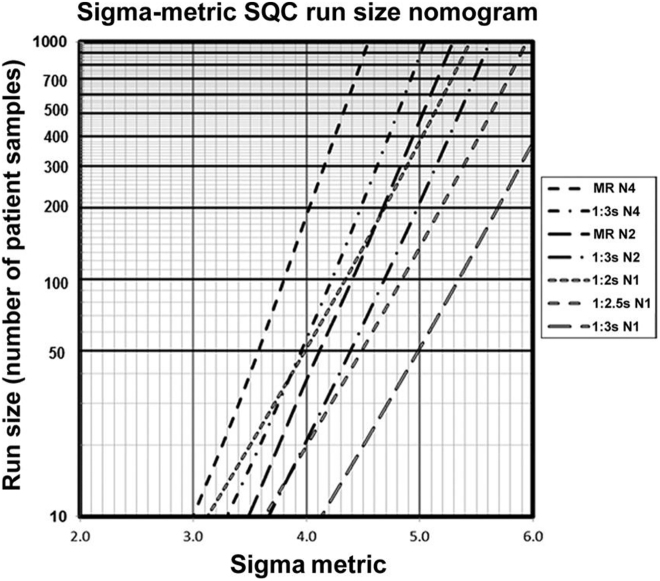
Nomogram based on σ metrics and work load to select the operative control rule. Obtained from Westgard JO et al. Clin Chem 2018;64:259–296 [52]. MR N4: multi-rule with 4 controls per run (1_3s_/2_2s_/R_4s_/4_1s_). MR N2: multi-rule with 2 controls per run (1_3s_/2_2s_/R_4s_). N: number of control results.

**Figure 2:**
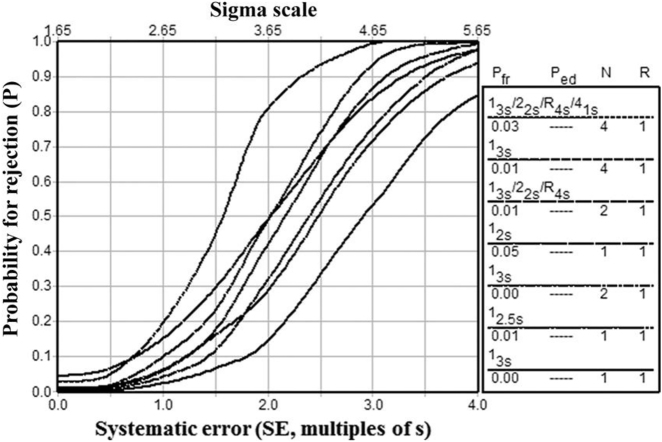
Power functions of the operative control rules included in the nomogram. Obtained from Westgard JO et al. Clin Chem 2018;64:259–296 [52]. p_fr_: probability for false rejection. p_ed_: probability for error detection. N: number of controls processed. R: number of analytical runs in which the operative control run is applied.

If there is not possible to have informatics support, it is extremely practical to use the nomogram proposed by Westgard et al. (Figure 1), where an operative control rule can be selected from the *σ* value and the number of patient samples to be tested [workload].The control rule can be translated to a power function graphs to identify its probability for error detection (p_de_) and for false rejection (p_fr_) (Figure 2) [52].

This model facilitates the use of different operative rules during the work day: strict rules for critical moments, such as calibration or instrument adjustment with maximum p_de_ and a more relaxed rule for monitoring analytical runs during the rest of the day with minimum p_fr_.

The strong point of this model, based on multi-rules amplified with estimating the frequency of control tests, is that it easy to be applied today because multi-rules are incorporated to most automatic analyzers and the mentioned Westgard nomogram is published [52].

### Considerations about POCT

Point of care testing (POCT) requires particular attention, because there are managed by non-laboratory personnel.

If qualitative or semi-quantitative tests are produced in simple devices [single strips, cassettes, cartridges], such as pregnancy tests or in house tests, simply positive and negative controls have to be tested and results should be in accordance.

When quantitative results are produced in more complex devices, such as HbA_1C_, blood glucose, as well as blood gases and complete blood count, it is necessary to use control materials in same way as it is done in the central laboratory [26] and the interchangeability of POCT results with the central laboratory should be assessed (ISO 22870) [53]. Also, testing patient’ samples in parallel with central lab is a good practice, when POCT location makes it possible. Venner et al. recommend testing a minimum of 10 positive patients and 10 negative patients in the initial verification of a POCT device; and five patients of each type in monitoring devices [54].

Various performance indicators for POCT have been proposed, which include extra-analytical steps, such difference between the number of tests considering the consumables used and the tests performed in POCT devices, percentage of the tests reported in LIS over the tests performed in POCT analyzers, percentage of non-identified operators. For the POCT analytical phase an example of indicator is the percentage or number of measurands with coefficient of variation within the analytical performance specification [55].

To evaluate staff training and competency and the fact that only qualified people use the POCT devices, a POCT indicator may be the percentage of tests performed by the POCT operator with the highest activity over all tests performed in every clinical setting.

When a relationship with central lab exists, it is important to implement the connectivity between laboratory informatics system and POCT data manager system, which allows to block or to impede releasing results from outside the laboratory when necessary.

### Present global view

Two surveys concerning IQC protocols were conducted by Sten Westgard to 700–900 laboratories from the five continents in 2017 and 2021. Questions were focused on two aspects: the IQC model used and the immediate management of control results control [56, 57]. Table 1 show the main results obtained in the two surveys.

**Table 1:** Worldwide surveys concerning the IQC model applied. Answers expressed in terms of percentage of laboratories.

IQC model	2017	2021
**Control limits**		

Laboratory standard deviation	63	58
Manufacturer control insert	43	57
Peer group standard deviation	20	24

**Operative control run**		

1_2s_	55	59
Multi-rule for all measurands	–	23
Multi-rule for some measurands	–	64

**Control sample origin**		

Instrument manufacturer	64	67
Third party, liquid, assayed	44	43
Third party, lyophilized	35	31
Third party, liquid, unassayed	30	20
Average of normals	11	14

**Frequency**		

Once per day	49	54
Several times per day	41	46
Staff criterion	38	38
According to patient risk	14	NR
Beginning and end work day	13	NR
Patient groups (i.e., every 100patients)	9	NR

NR, not requested.

**Table 2:** Worldwide surveys concerning the immediate management after an out-of-control warning. Answers expressed in terms of percentage of laboratories.

Immediate management	2017	2021
**Out-of-control measurement procedure**		

To search for reasons before repeating patient samples	78	79
To repeat control sample	78	68
To prepare a new control sample	64	55
To recalibrate the instrument	16	20
To immediately notify the manufacturer	2	4

**Retesting patient samples**		

Only determined groups	33	31
All daily patients	32	33
Only abnormal results	20	24
Only those near the control failed	13	14

**Releasing patient results when control failed**		

Never	54	48
Few times (<10/month)	30	30

IQC model used implemented:

– IQC procedures should be planned to verify attainment of the quality required for intended use, accounting for the observed bias and precision of the method and the rejection characteristics of the SQC procedure.
– All control limits are statistical limits, but the particular decision rules and number of measurements can be selected to ensure the desired quality is achieved. If the QC selection/planning activity neglects to define the quality required for intended use, then the IQC procedure only ensures an unknown arbitrary level of quality.
– The most widely control material used is that coming from the instrument manufacturer, whereas using third-party control is the recommended by the accreditation norm ISO 15189 [32].
– The operative control rule most frequently used is still the 1_2s_, which generally has high probability for false rejection thus being considered inefficient.
– It does not seem evident that many laboratories adjust the frequency of control testing to its workload, not being aware to the risk management of their patients. This item should be considered in the immediate future.
– About 45% of laboratories are aware of the ISO 15189 norm or of the country regulation, which is considered to be a very positive aspect.

The immediate management after an out-of-control measurement procedure, shown in Table 2, illustrates the following situation:

– A general tendency to look at the failure reasons before repeating the control test or to prepare a new control sample is observed, being positive attitude. Fortunately, very few laboratories simply call the manufacturer immediately, which is considered to be few adequate.
– Repeating patient samples nearer to the control that failed or patient results with abnormal results is the most widely used option and, also, the most adequate. It is not considered to be appropriate to repeat all patient samples of the affected run, at least if no more control results had failed too; there is still 30% of lab anchored in this usage.
– It is a surprise that half of the surveyed laboratories release results when the control failed, a practice that should not be used anymore.

Concerning performance specifications, a survey was made in Spain in 2015 in Spain to 1738 laboratories [58]. Half of the participants [47%] used specifications derived from biological variation, 33% from the state of the art stated by the Spanish consensus for minimum quality specifications [59], 5% from clinicians’ opinions, 3% from regulations of other countries such as CLIA of USA [60] and Rilibäck of Germany [61] and the remaining laboratories used other criteria.

On the other hand, it is necessary to implement a long-term management of data given by IQC, consistent in to periodically (i.e. monthly) evaluate performance and to compare with the specifications admitted by the laboratory. If they are not attached, it has to verify if the IQC has been rigorously followed, if out-of-control analytical runs have been erroneously accepted, if maintenance of equipment has been correct, if technician has been properly instructed, etc. [62, 63]

As Sten Westgard concludes in his 2021 survey, labs should change and adopt better IQC protocols; otherwise, they put on risk its own viability and, worse, put patients on risk because of their poor lab reports.

## Conclusions

The strong points revealed from the light of this revision are:

– Well established IQC protocols do exist, to monitor analytical performance and to detect changes of systematic error, using control samples and also sing patient samples.
– Third-party non commutable control material exists, to produce good IQC and to provide information from other labs using same measurement method and same control material.
– Data on biological variation to establish control limits are highly reliable and exist for a great number of measurands.
– The workload and the impact of an analytical failure on the information concerning patient status is easy to be known and should be applied in the IQC protocol.

The weak points to be eliminated are:

– Using the 1_2s_ operative control rule because its high p_fr_.
– Using control limits simply based on statistics rather than control rules derived from quality specifications.
– Repeating control tests without have first searched the reasons for failure.
– Repeating all patient samples without to check where the failure was produced.
– Releasing patient results when a failure was produced.

The projection for future is

– To use third-party control materials.
– To implement IQC based on patient samples when no stable control material is available.
– To use analytical performance specifications based on biological variation.
– To encourage external quality assessment organizers to provide commutable controls and to push laboratories to have freezers to maintain them at −80 °C.
